# Multiple Antimicrobial Resistance in Methicillin-Resistant *Staphylococcus sciuri* Group Isolates from Wild Ungulates in Spain

**DOI:** 10.3390/antibiotics10080920

**Published:** 2021-07-28

**Authors:** Joaquín Rey Pérez, Laura Zálama Rosa, Alfredo García Sánchez, Javier Hermoso de Mendoza Salcedo, Juan Manuel Alonso Rodríguez, Rosario Cerrato Horrillo, Sofía Gabriela Zurita, María Gil Molino

**Affiliations:** 1 Unidad de Patología Infecciosa y Epidemiología, Facultad de Veterinaria, Universidad de Extremadura, 10003 Cáceres, Spain; laura.zalama94@gmail.com (L.Z.R.); jhermoso@unex.es (J.H.d.M.S.); jmalonso@unex.es (J.M.A.R.); sofigz32@gmail.com (S.G.Z.); magilmo84@gmail.com (M.G.M.); 2 Área de Producción Animal, CICYTEX-La Orden, 06187 Badajoz, Spain; fredgarsa@gmail.com; 3 Innovación en Gestión y Conservación de Ungulados S.L., 10004 Cáceres, Spain; rosario@ingulados.com

**Keywords:** antimicrobial resistance, methicillin-resistant, red deer, *Staphylococcus sciuri*, wild boar, wildlife, One Health

## Abstract

The aim of this study was to investigate the presence of methicillin-resistant *Staphylococcus* (MRS) strains in non-managed wild ungulates present in a typical Mediterranean forest in Spain. For this purpose, nasal swabs were obtained from 139 animals: 90 wild boar (*Sus scrofa*), 42 red deer (*Cervus elaphus*) and 7 fallow deer (*Dama dama*), which were subsequently pre-enriched in BHI+ NaCl (6.5%) (24 h/37 °C), and then seeded in Columbia blood agar (24 h/37 °C)). The presence of the *mecA* gene was investigated by PCR, first from the confluent and then from individual colonies. A total of 10 *mecA*+ colonies were obtained of which only seven showed phenotypic resistance to oxacillin/cefoxitin (methicillin resistance). All MRS strains belonged to the *Staphylococcus sciuri* group. Methicillin-resistant *Staphylococcus aureus* (MRSA) was not detected. In addition, a significant number of MRS strains showed resistance to other antimicrobials, mainly β-lactam (7/7), gentamicin (7/7), fusidic acid (6/7) and quinupristin-dalfopristin (6/7), showing an irregular correlation with their coding genes. The genetic profiles grouped the seven strains obtained according to the bacterial species but not in relation to the animal source or the geographical place of origin. The presence of SCC*mec* type III, common to animals and humans, has been detected in three of the strains obtained. In conclusion, the study reveals that the wild ungulates investigated play a role as potential reservoirs of multi-resistant strains of MRS. Such strains, due to their characteristics, can be easily transferred to other wild or domestic animal species and ultimately to humans through their products.

## 1. Introduction

Antimicrobial resistance is widespread in animal reservoirs. This resistance has been observed mainly in domestic animals, due to the selective pressure exerted by the frequent and non-specific use of antimicrobials for preventive or curative purposes, and, mainly in pigs and poultry, as growth promoters as well. This resistance is not limited to domestic animals but has extended to other wild species sharing the same habitats and resources [1]. Such is the case with wild boar and, to a lesser extent, other wild ungulates in the Mediterranean ecosystem. These species, due to their abilities of toughness, prolificacy and dispersion, accumulate the resistance and virulence genes circulating in a given ecosystem [2]. This is facilitated by the fact that these genes are frequently encoded in mobile genetic elements (plasmids, chromosomal cassettes, transposons). These elements are easily transferred horizontally between micro-organisms, regardless of the pathogenic or non-pathogenic nature of their recipients [3]. A paradigmatic example of this phenomena is what happened with methicillin resistance (MR) in the genus *Staphylococcus*. This resistance, first noted in 1961 in *Staphylococcus aureus* (MRSA) isolated from humans in the UK [4], spread rapidly to other countries and environments, eventually colonizing domestic and wild animals. The presence of this resistance is related to the acquisition of the *mecA* gene as part of a mobile genetic element called Staphylococcal Cassette Chromosome *mec* (SCC*mec*) [5]. SCC*mec* is easily transferable between populations, which confers resistance to almost all β-lactams and occasionally to other groups of antimicrobials by the synthesis of a low-affinity PBP protein (PBP2a) [6]. Due to its severe clinical and therapeutic implications, most studies referring to this resistance have focused on coagulase-positive *Staphylococcus* (CoPS), especially MRSA isolated from humans and domestic species. In contrast, only a few studies have investigated its presence in other species of coagulase-negative *Staphylococcus* (MRCoNS), and even fewer have focused on wild species. Thus, the importance that these species may have as a long-term reservoir of these and other resistance genes is underestimated.

Therefore, the purpose of our work has been to investigate the presence of methicillin-resistant *Staphylococcus* (MRS) in wild ungulates, to identify the species, to determine the associated phenotypic and genotypic patterns of resistance and, finally, to clonally investigate the obtained isolates and their SCC*mec* type, thus determining the significance of these species as reservoirs and their potential as a disseminator of such strains.

## 2. Results

### 2.1. Prevalence and Species of MRS

From the 139 nasal swabs processed (wild boar: 90; red deer: 42 and fallow deer: 7), MRS were obtained in seven of them (5.03%; 95%, CI: 1.4–8.7): five in wild boar and two in red deer, with prevalences of 5.55% (95%, CI: 0.8–10.3) and 4.76% (95%, CI: 0.1–11.2), respectively. None were detected in fallow deer. Isolates 115, 121 and 127, although carrying the *mecA* gene, did not develop the phenotypic profile of oxacillin/cefoxitin resistance and are therefore not considered MRS.

Among the seven MRS isolates obtained, three different *Staphylococcus* species were identified, all of them belonging to the *Staphylococcus sciuri* group (SSG): *S. vitulinus* (*n* = 4), *S. fleuretti* (*n* = 2) and *S. sciuri* (*n* = 1). The three species described were isolated from wild boar: *S. vitulinus* (*n* = 2), *S. fleuretti* (*n* = 2) and *S. sciuri* (*n* = 1); while only *S. vitulinus* was identified in red deer (*n* = 2). MRSA was not detected (Table 1).

### 2.2. Antimicrobial Resistance Patterns

**Figure 1:**
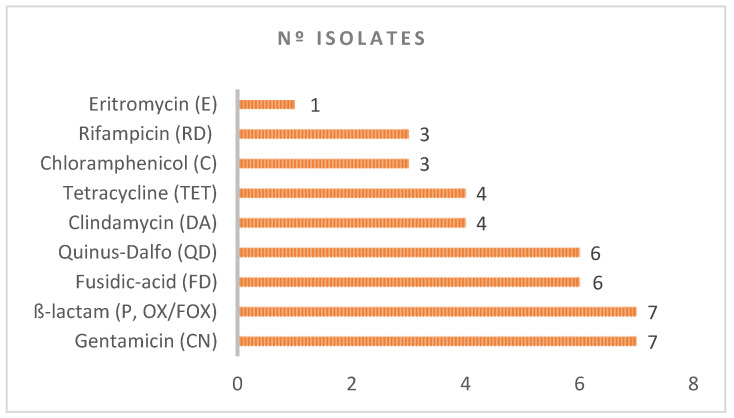
Antimicrobial resistance associated with the methicillin-resistant isolates.

MRS isolates were frequently associated with other types of resistance. Thus, we have also observed resistance to aminoglycosides (CN): seven isolates; β-lactam (P, FOX, OX): seven isolates; fusidic acid (FD): six isolates; quinupristin-dalfopristin (QD): six isolates; clindamycin (DA): four isolates; tetracycline (TET): four isolates. Other antimicrobials (choramphenicol, rifampicin and erythromycin) exhibited lower antimicrobial resistance (three, three and one isolates, respectively), and the others none (trimethoprim–sulphamethoxazole, linezolid) (Table 1 and Figure 1).

We have detected a good correlation between the coding genes detected and the phenotypic resistances observed for *TetK/M* (TET) (4/4) and *mecA* (P, FOX and OX) (7.4 and 7/7, respectively), but it was low for *aacA-AphD* (CN) (2/7) and there was none for *blaZ* (P) (0/7) or *FusB/FusC* (FD) (0/6) (Table 1).

### 2.3. Types of SCCmec and DNA Macrorestriction Profiles

**Figure 2:**
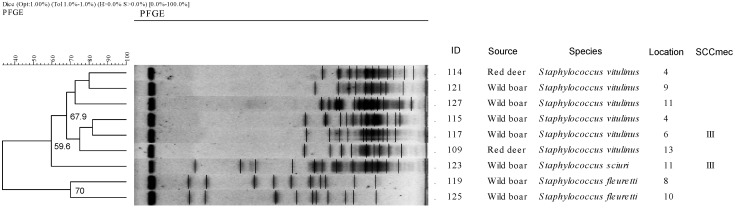
Dendogram based on PFGE macrorestriction pattern of *mecA*-positive *Staphyloccus* isolates. Dendogram showing 9 different profiles further divided into three clusters corresponding to the three staphylococcus species. The scale at the top indicates the similarity indices (in percentages). Isolate nº 102 (SCC*mec* type III) was non-typeable.

In our study, we detected the presence of SCC*mec* type III in three strains (102, 117 and 123), isolated from wild boar and belonging to the species *S. vitulinus* and *S. sciuri*, while the rest of the strains were non-typified (NT) (Figure 2).

On the other hand, a total of nine different macrorestriction profiles and one non-typeable sample (102) were obtained in the 10 isolates processed by PFGE (Figure 2). The profiles obtained show grouping by bacterial species, defining three clusters with a similarity of more than 59.6%. Thus, cluster 1, with a similarity of 67.9%, grouped the six profiles derived from the isolates of the species *S. vitulinus* obtained in wild boar and deer; cluster 2 included the only isolate obtained from *S. sciuri* in wild boar; and finally, cluster 3, with a similarity of 70%, included the two isolates of the species *S. fleuretti* obtained in wild boar. Except for isolates 114 and 115, which came from geographical area 4, and 123 and 127, which came from geographical area 11, the rest of the isolates were obtained from separate geographical locations. No major similarity is observed in terms of animal species or place of origin of the isolates. Interestingly, all *S. vitulinus* strains had a macrorestriction profile characterized by the presence of small molecular fragments while the other species (*S. sciuri* and *S. fleuretti*) had a more heterogeneous profile.

## 3. Discussion

In general, wild species, and specifically wild ungulates, are not considered to be good reservoirs for MRSA [7,8,9], and, in fact, in our study, we have not detected their presence in any of the animals investigated. This scarcity of isolates has been a constant in most research conducted on wild ungulates including those conducted in the Iberian Peninsula, confirming their low prevalence, with values never exceeding 1% [9,10].

This low prevalence of MRSA in wild species markedly contrasts with the values observed in domestic species, mainly in ruminants and pigs, which are often located in areas close to wild species. The pig, due to its genetic proximity to the wild boar, is of particular interest for our study, allowing us to analyze the importance that certain factors, mainly environmental, may have in the development of this resistance. The prevalence is particularly high (25–40%) in intensive pig farming, with high zootechnical specialization [11] while it is dramatically reduced in less technified pig farms [12]. In this sense, Meenken in Germany [13] compared the frequency of MRSA findings in conventional pig herds with those of organic pig herds and wild boars, finding a higher prevalence of MRSA in conventional farms (18%), compared to organic (1%) and wild boars in which no MRSA was detected. Therefore, the differences observed in the prevalence of MRSA between pigs and wild boar seem to be related more to differences in the use of antimicrobials, animal densities and production stress, than to factors intrinsic to the animals themselves.

All *mecA*-positive strains detected in our study are CoNS belonging to the *Staphylococcus sciuri* group (SSG). This group includes the species *S. sciuri*, *S. vitulinus*, *S. lentus*, *S. fleurettii* and *S. stepanovicci* [14]. They are novobiocin-resistant *Staphylococcus*, which are part of the microbiota of many warm-blooded animals [15], and only occasionally producing pathological processes, so they are considered more opportunistic than primary pathogens. This species is also highly resistant to the environment, having ample metabolic resources as well [16]. Due to its ubiquitous nature, its great environmental resistance and extraordinary ability for genetic exchange, this species tends to accumulate in its genome a significant proportion of the resistance and virulence genes circulating in a given ecosystem. Thus, it becomes a reservoir and a source for dissemination of these genes for the rest of the bacteria [17].

MR associated with SSG and other CoNS has been widely documented in an independent fashion, or, more frequently, associated with other types of resistance in livestock and poultry [18], animal products [19,20], horses [21], pets [22] and the environment (dust, manure and litter) [23,24]. Nevertheless, there are very few studies on this resistance in CoNS in wild species. In a large study focusing on the CoNS from wild boar in Spain [25], the presence of MR was found in 1.8% of the animals investigated (4/371). It was associated with the species *S. fleuretti* (2), *S. vitulinus* (1) and *S. haemolyticus* (1), with a joint presence of resistance to tetracycline, clindamycin and erythromycin in some strains. In our study, we have found an overall MR prevalence of 5.03%, with species-specific values of 5.55% for wild boar and 4.76% for deer. Furthermore, half of the isolates obtained showed joint resistance to at least five different groups of antimicrobials. These are much higher values than those detected to date and are increasingly reminiscent of those detected in domestic animals in the same areas [26].

Regarding the types of resistance observed, some correspond to antimicrobials used frequently in human and veterinary medicine, mainly β-lactams, aminoglycosides and tetracyclines [26].

The location of all the genes responsible for these resistances in mobile elements of different natures (plasmids, transposons, SCC) would greatly facilitate their horizontal exchange and the spread of these resistances between different populations [3]. However, it is interesting to note that these resistances, at first mainly restricted to the sphere of domestic animals, their environment, their products or their waste, are gradually spreading to wild species that have theoretically not had the opportunity for previous direct contact with these antibiotics. Other types of resistances have been much less common (chloramphenicol, erythromicin) or non-existent (trimethoprim/sulphamethoxazole, linezolid).

We found an irregular correlation between the phenotypic characteristics observed and their coding genes. Thus, this correlation was total between TET and *tetK/M* (4/4) and slight between CN and *aacA-AphD* (2/9), but null between FD and *fusA/C* (0/7). These apparent discrepancies are frequent among CoNS because the genes responsible for their resistance are not always perfectly characterized [27], and there are frequent mutation events which make their detection difficult. In relation to β-lactams, we found an interesting fact: although all isolates harbored the *mecA* gene, two different phenotypic patterns were evident: one resistant to the β-lactams tested (P, OX/FOX) (7/10), and another sensitive (3/10). This circumstance could be related to the existence in *S. sciuri, S. vitulinus* and *S. fleurettii* of a homologue to the *mecA* gene, with which they share a 79.5% nucleotide sequence identity, not located in SCC*mec* but in the chromosomal DNA, which does not confer resistance to β-lactams [28]. In this study, therefore, we could have detected two different types of *mecA* genes: the one present in the SSG, which would be present in all the isolates obtained and which does not confer resistance; and the one characteristic of MRSA, which would be present in seven of the isolates obtained and which is associated with the resistance observed. This circumstance, however, will have to be confirmed later with more specific tests that allow us to discriminate between the two genes.

In our study, we have found the presence of SCC*mec* type III in three of the isolates obtained from wild boar. The rest of the isolates (*n* = 7) have been shown as non-typeable (NT). Several studies show that SCC*mec* type III is the most frequently identified among the CoNS in animals [29,30] and, together with type II, harbors additional resistance genes. Furthermore, this type of SCC*mec* has been frequently isolated in pigs and ruminants [31]. These are species with which wild boar share habitats and resources in the Mediterranean ecosystem, allowing an easy exchange of this element. In this way, the CoNS present in wild ungulates become reservoirs of the *mecA* gene for domestic species. Then, they can ultimately be transferred to humans through their products. As is known, SCC*mec* type III is one of the most prevalent among hospital-acquired methicillin-resistant *Staphylococcus aureus* (HA-MRSA) [32]. The high percentage of NT strains obtained in this work is remarkable. This circumstance is frequent among CoNS of animal origin, and especially frequent among SSG. The reason is the great divergences present in the composition of the *ccr* and *mec* complexes with the development of SCC*mec* that are not well characterized [30].

Regarding the genetic relationship of the isolates obtained, we have observed a certain homogeneity in the isolates according to their bacterial species, regardless of the animal species from which they were isolated or their geographical origin. This homogeneity is preserved even in isolates obtained from different species geographically separated from each other (isolates 114 and 121, from location 4 and 8, and isolates 115 and 117, from location 4 and 5) (similarity superior to 80%).

The presence of such multi-resistant strains in wild boar is not coincidental. What the SSG represents for the rest of the micro-organisms in terms of resistance, dispersion and gene accumulation capacity is equivalent to what the wild boar represents for the rest of the higher animals in the Mediterranean ecosystem. In recent years, wild boar have experienced a notable increase in populations. This is due to intrinsic factors (prolificacy, hardiness, omnivorous/carrion-eating nature); ecological factors (lack of natural predators, especially the wolf and the bear); and economic/social factors (crisis of traditional livestock farming/agriculture with abandonment of rural areas and increase in uncultivated areas) [2]. All of these factors facilitate this species to multiply and expand uncontrollably to peri-urban areas. There, it easily interacts with humans and different domestic species, by gaining access to the waste they generate. In this situation, due to their great toughness, wild boar progressively accumulate the micro-organisms and the genes resulting from these contacts. In this sense, it must be said that although domestic species are regularly subjected to sanitary control, this is not the case with wild species. In these circumstances, wild boar become a long-lasting reservoir of all the bacteria and genes circulating in each ecosystem and a magnificent bio-indicator of the ecosystem.

Under a One Health strategy, non-managed wild ungulate populations living in interface areas with livestock are excellent indicators of antimicrobial resistance. Thus, they should be monitored as a first step to design strategies aiming to hinder their role as reservoirs and disseminators of pathogenic agents and of antimicrobial resistance potentially dangerous for other species, including humans.

## 4. Materials and Methods

### 4.1. Game States

**Figure 3:**
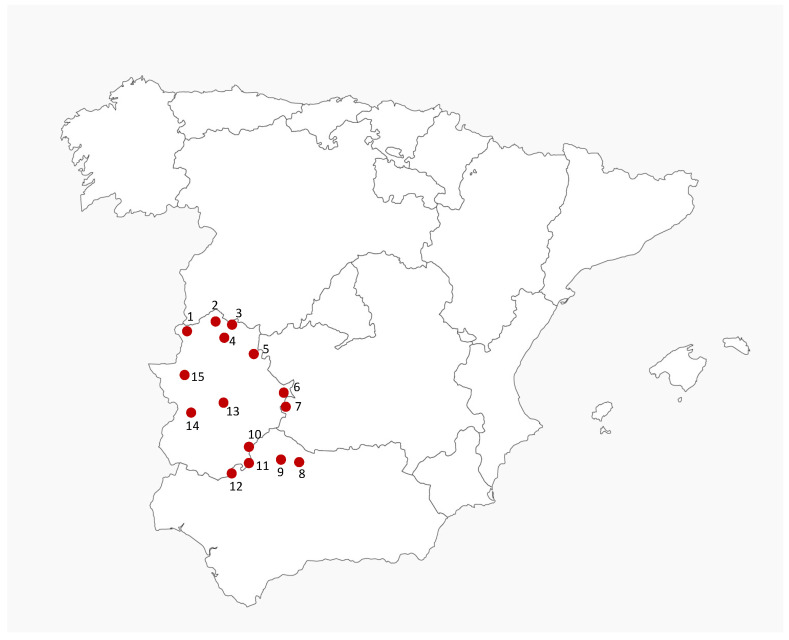
Physical map of Spain showing the location of the different game estates investigated in this study. Numbers identify the different geographical locations of samples.

The animals in this study came from 15 different hunting actions carried out on game estates in central western Spain. This area is characterized by a typical Mediterranean ecosystem called “Dehesa”, where wild ungulates share habitats and resources with domestic species, especially pigs and ruminants (Figure 3). These animals had never been treated with antibiotics.

### 4.2. Sample Collection

Nasal swabs were obtained from a total of 139 animals: 90 wild boar (*Sus scrofa*), 42 red deer (*Cervus elaphus*) and 7 fallow deer (*Dama dama*) at the veterinary inspection meeting after the hunt. The samples, after being collected, were kept in a Stuart–Amies medium (DeltaLab), refrigerated at 5ºC and transferred to the laboratory, where they were processed in within 24 h.

### 4.3. Bacterial Culture, Detection of the mecA Gene and Conservation

The samples were pre-enriched in brain heart infusion (BHI) broth (Oxoid, Madrid, Spain) supplemented with NaCl (6.5%) and incubated at 37 °C/24 h. Aliquots of 100 µL were cultured on Columbia blood agar (Oxoid) in aerobic conditions for 24 h at 37 °C.

The presence of the *mecA* gene was verified by means of PCR, first on the confluent growth of each plate, and subsequently, if positive, from individual re-isolated colonies. This procedure was repeated until a positive individual colony was obtained. For DNA extraction, colonies were suspended in 500 µL of sterile distilled water and heated at 99 °C for 10 min. After centrifugation at 10,000 rpm for 5 min, supernatant was used as a template. Amplification of the *mecA* gene for the detection of methicillin resistance was performed in a 25 µL mixture containing 12.5 µL of 2X FastGene^®^ Optima HotStar (Nippon Genetics, Düren, Germany), primers *mecA1*(5′-GTTGTAGTTGTCGGGTTTGG-3′) and *mecA2*(5′-CGGACCTTCAGTCATTTCTAC-3 [33] in a final concentration of 0.5 mM each primer, and 5 µL of template. Thermocycling conditions were set at 94 °C for 5 min, followed by 30 cycles of 95 °C 45 s, 55 °C 20 s, 72 °C 50 s, followed by a 5 min incubation at 72 °C and holding at 5 °C. Amplification produced a band with a molecular size of 161 bp. *mecA*-positive colonies were stored at −70 °C in freezer vials pending further analysis.

### 4.4. Species Identification

Based on the information provided by Gram staining and catalase and oxidase biochemical testing, those colonies compatible with *Staphylococcus* spp. were identified using matrix-assisted laser desorption/ionization time-of-flight mass spectrometry (MALDI-TOF MS) [34] considering Brukers’ cut-off value for reliability (LogScore > 1.70).

### 4.5. Antimicrobial Resistance and Identification of Antimicrobial Resistance Genes

Antimicrobial susceptibility testing on 13 antimicrobials was performed by the disc diffusion method [35] for all the recovered isolates. A panel of 13 antimicrobials representing different classes was selected, all of them with a background history of antimicrobial resistance in the genus *Staphylococcus*. The following discs (Oxoid^®^) were used: penicillin (P) (1 unit); cefoxitin (FOX) (30 μg); oxacillin (OX) (1 μg); tetracycline (T) (30 μg); chloramphenicol (C) (30 μg); gentamicin (CN) (10 μg); erythromycin (E) (15 μg); clindamycin (DA) (2 μg); quinupristin/dalfopristin (QD) (15 μg); linezolid (LZD) (10 μg); rifampicin (RD) (5 μg); trimethoprim/sulphamethoxazole (SXT) (1.25/23.75 μg) and fusidic acid (FD) (10 μg). *Staphylococcus aureus* ATCC 29213 and NCTC 12493 were used as control strains.

The presence of antimicrobial resistance genes was investigated by specific PCRs for genes *blaZ* [36], *aacA-AphD*, *TetK*, *tetM* [37], *FusB* and *FusC* [38], representing the most frequently observed antimicrobial resistance types in phenotyping studies.

### 4.6. Typing of Staphylococcal Cassette Chromosome mec (SCCmec)

The characterization of the SCC*mec* types was carried out by performing four multiplex PCRs: M-PCR 1 for identifying *mecA* and *ccr* gene complex types; M-PCR 2 for identifying *mec* gene complex classes A, B and C2; M-PCR 3 for amplification of ORFs in the J1 region of type IV SCC*mec* and M-PCR 4 for amplification of ORFs in the J1 region of type II SCC*mec,* which allowed us to identify the main SCC*mec* (types I-XIII) [39]. Strains NCTC10442 (type I), N315 (type II), 85/2082 (type III), JCSC4744 (type IVa), JCSC2172 (type IVb), JCSC4788 (type IVc), JCSC4469 (type IVd), WIS (type V), HDE288 (type VI), JCSC6082 (type VII), C10682 (type VIII), JCSC6943 (type IX), JCSC6945 (type X) and LGA251 (type XI) were used as control strains.

### 4.7. Phylogenetic Analysis Using Pulsed-Field Gel Electrophoresis (PFGE)

Determination of the genetic relationship between isolates was performed by macrorestriction with *Sma*I followed by pulsed field gel electrophoresis (PFGE) (Chef-Mapper XA. BioRad^®^, Hercules, CA, USA) according to the PulseNet protocol [40]. The different PFGE profiles (PFPs) were analyzed by InfoQuest FP Software (Bio-Rad v. 4.5, Hercules, CA, USA). A dendrogram was derived by the unweighted pair group method using the arithmetic average (UPGMA) and based on the Dice coefficient at band optimization of 1% and 1% band position tolerance. 

## 5. Conclusions

In the Spanish Mediterranean ecosystem, wild boar and, to a lesser extent, red deer, carry MRCoNS strains belonging to the SSG group. These strains also harbor resistance to other antimicrobials frequently used in livestock reared in the same environment. The features of the mobile genetic elements that encode these resistances, in our work, SCC*mec* type III, greatly facilitate the exchange of these resistances between different bacterial and animal species, both wild and domestic, and can eventually pass to humans through direct contact or through the consumption of animal products.

## Figures and Tables

**Table 1:** Identification, origin and antibiotic resistance characteristics of the *mecA*-positive staphylococci isolates.

ID	Species	Source	Phenotypic Resistance	Genotype
102	*S. vitulinus*	Wild boar	P, FOX, OX, TET, C, CN, DA, QD, RD, FD	*mecA, aacA-AphD, tetK, tetM*
114	*S. vitulinus*	Red deer	P, FOX, OX, TET, C, CN, DA, QD, RD, FD	*mecA, aacA-AphD, tetK, tetM*
119	*S. fleuretti*	Wild boar	P, FOX, OX, TET, C, CN, DA, QD, FD	*mecA, tetM*
117	*S. vitulinus*	Wild boar	P, FOX, OX, TET, CN, DA, QD, RD, FD	*mecA, tetM*
123	*S. sciuri*	Wild boar	P, OX, CN, E, QD, FD	*mecA*
125	*S. fleuretti*	Wild boar	P, OX, CN, QD, FD	*mecA*
109	*S. vitulinus*	Red deer	P, OX, CN	*mecA*
127	*S. vitulinus*	Wild boar	FD	*mecA*
121	*S. vitulinus*	Wild boar	CN	*mecA*
115	*S. vitulinus*	Wild boar	CN	*mecA*

Although all isolates have the *mecA* gene, the phenotypic resistance profile shows that only isolates 102, 109, 114, 117, 119, 123 and 125 are methicillin resistant. P: Penicillin, FOX: Cefoxitin, OX: Oxacillin, TET: Tetracycline, C: Chloramphenicol, CN: Gentamicin DA: Clindamycin, QD: Quinupristin-dalfopristin, E: Erythromycin, RD: Rifampin, FD: Fusidic acid.

## Data Availability

The data presented in this study are available upon request.
